# An Augmented Negative Force-Frequency Relationship and Slowed Mechanical Restitution Are Associated With Increased Susceptibility to Drug-Induced Torsade de Pointes Arrhythmias in the Chronic Atrioventricular Block Dog

**DOI:** 10.3389/fphys.2018.01086

**Published:** 2018-08-08

**Authors:** David J. Sprenkeler, Alexandre Bossu, Jet D. M. Beekman, Marieke Schoenmakers, Marc A. Vos

**Affiliations:** Department of Medical Physiology, Division of Heart and Lungs, University Medical Center Utrecht, Utrecht, Netherlands

**Keywords:** force-frequency relationship, mechanical restitution, post-extrasystolic potentiation, Torsade de Pointes, contractile remodeling, chronic AV-block dog

## Abstract

**Introduction:** In the chronic AV-block (CAVB) dog model, structural, contractile, and electrical remodeling occur, which predispose the heart to dofetilide-induced Torsade de Pointes (TdP) arrhythmias. Previous studies found a relation between electrical remodeling and inducibility of TdP, while structural remodeling is not a prerequisite for arrhythmogenesis. In this study, we prospectively assessed the relation between *in vivo* markers of contractile remodeling and TdP inducibility.

**Methods:** In 18 anesthetized dogs, the maximal first derivative of left ventricular pressure (LV dP/dt_max_) was assessed at acute AV-block (AAVB) and after 2 weeks of chronic AV-block (CAVB2). Using pacing protocols, three markers of contractile remodeling, i.e., force-frequency relationship (FFR), mechanical restitution (MR), and post-extrasystolic potentiation (PESP) were determined. Infusion of dofetilide (0.025 mg/kg in 5 min) was used to test for TdP inducibility.

**Results:** After infusion of dofetilide, 1/18 dogs and 12/18 were susceptible to TdP-arrhythmias at AAVB and CAVB2, respectively (*p* = 0.001). The inducible dogs at CAVB2 showed augmented contractility at a CL of 1200 ms (2354 ± 168 mmHg/s in inducible dogs versus 1091 ± 59 mmHg/s in non-inducible dogs, *p* < 0.001) with a negative FFR, while the non-inducible dogs retained their positive FFR. The time constant (TC) of the MR curve was significantly higher in the inducible dogs (158 ± 7 ms versus 97 ± 8 ms, *p* < 0.0001). Furthermore, a linear correlation was found between a weighted score of the number and severity of arrhythmias and contractile parameters, i.e., contractility at CL of 1200 ms (*r* = 0.73, *p* = 0.002), the slope of the FFR (*r* = -0.58, *p* = 0.01) and the TC of MR (*r* = 0.66, *p* = 0.003). Thus, more severe arrhythmias were seen in dogs with the most pronounced contractile remodeling.

**Conclusion:** Contractile remodeling is concomitantly observed with susceptibility to dofetilide-induced TdP-arrhythmias. The inducible dogs show augmented contractile remodeling compared to non-inducible dogs, as seen by a negative FFR, higher maximal response of MR and PESP and slowed MR kinetics. These altered contractility parameters could reflect disrupted Ca^2+^ handling and Ca^2+^-overload, which predispose the heart to delayed- and early afterdepolarizations that could trigger TdP-arrhythmias.

## Introduction

Despite advances in treatment and prevention strategies, sudden cardiac death caused by ventricular arrhythmias remains a common cause of death in patients with heart failure or compensated hypertrophy (Tomaselli and Zipes, 2004). In response to certain stressors, these patients exhibit ventricular remodeling, an adaptive process that initially helps to maintain normal cardiac function, but eventually becomes maladaptive, causing electrical instability and an increased risk of life-threatening ventricular arrhythmias (Konstam et al., 2011).

The chronic AV-block (CAVB) dog model is a widely used animal model to study ventricular remodeling and its relation with ventricular arrhythmias (Oros et al., 2008; Zhou et al., 2008; Wada et al., 2016). In this model, creation of third degree AV-block results in bradycardia and volume overload. To compensate for the resulting drop in cardiac output, ventricular remodeling occurs (Volders et al., 1998; Vos et al., 1998). This remodeling process reduces the so called “repolarization reserve,” the ability of the heart to cope with stressors on repolarization (Roden, 1998). When repolarization is then further challenged by anesthesia and administration of a pro-arrhythmic drug such as dofetilide, early afterdepolarizations (EADs), ectopic beats, and TdP arrhythmias start to occur (Dunnink et al., 2010).

Ventricular remodeling is a complex process and can be divided into multiple components, such as structural, electrical, and contractile remodeling. However, the contribution of each of the three different components to arrhythmogenesis is not fully elucidated. Previous studies have shown that electrical remodeling is an important contributor to the susceptibility of TdP, (Vos et al., 1998) while structural remodeling is not a prerequisite (Schoenmakers et al., 2003). Electrical remodeling, which is reflected by prolongation of the action potential duration (APD) and increased spatial and temporal dispersion of repolarization, (Verduyn et al., 1997a,b; Thomsen et al., 2006, 2007) develops in synchrony with TdP-inducibility: both are present after 2 weeks of CAVB (Vos et al., 1998; Schoenmakers et al., 2003). Structural remodeling, on the other hand, follows a much slower path and is fully present after 16 weeks of CAVB (Schoenmakers et al., 2003; Peschar et al., 2004; Donker et al., 2005).

Yet, the time course of contractile remodeling and its relation to TdP-inducibility is less well described. Contractile adaptations, as a result of changes in Ca^2+^ handling, can be measured *in vivo* by three physiological mechanisms of the heart: force-frequency relationship (FFR), mechanical restitution (MR) and post-extrasystolic potentiation (PESP). The FFR accounts for potentiation of contractility when heart rate is increased. MR and PESP relate to changes in contractile force of the extrasystolic beat and post-extrasystolic beat, respectively, when a basic stimulation train is interrupted with extrastimuli. MR represents the restoration of contractile force of the extrasystolic beat when the CI of the extrastimulus is lengthened. PESP displays the opposite behavior: when CI is shortened, the contractility of the post-extrasystolic beat is enhanced. Thus, the earlier an extrasystole occurs, the lower the contractility of the extrasystolic beat and the higher the contractility of the post-extraystolic beat.

de Groot et al. (2000) showed that FFR and PESP are altered in the CAVB-dogs after 6 weeks of remodeling and that these contractile parameters are associated with delayed afterdepolarizations (DADs) *in vivo*. Furthermore, in isolated cardiomyocytes from CAVB-dogs, Ca^2+^-overload of the sarcoplasmic reticulum (SR) can result in spontaneous Ca^2+^ release, which triggers EADs/DADs (Sipido, 2006). However, it is unknown whether these macroscopic measures of Ca^2+^-handling (FFR, MR, PESP) also reflect the propensity for sustained TdP-arrhythmias. If contractility and arrhythmogenesis are intertwined, contractile parameters could function as markers of pro-arrhythmia and might eventually help to identify the patient at risk for life-threatening arrhythmias. Therefore, we aimed to investigate the relation between *in vivo* contractility measures and susceptibility to TdP-arrhythmias in the CAVB dog. We assessed these measures after 2 weeks of remodeling, since CAVB-dogs are susceptible to TdP-arrhythmias from that moment onward.

## Materials and Methods

Animal handling was in accordance with the “Directive 2010/63/EU of the European Parliament and of the Council of 22 September 2010 on the protection of animals used for scientific purposes” and the Dutch law, laid down in the Experiments on Animals Act. All experiments were performed with approval of the Central Authority for Scientific Procedures on Animals (CCD), registered as project AVD115002016531.

### Animal Preparation

Eighteen adult purpose-bred mongrel dogs of either sex (Marshal, United States; 5 females, 13 males; bodyweight 26 ± 0.63 kg) were included. Experiments were performed under general anesthesia with mechanical ventilation at 12 breaths/min. The dogs were premedicated with a mixture of 0.5 mg/kg methadone, 0.5 mg/kg vetranquil and 0.02 mg/kg atropine i.m.. General anesthesia was induced with 25 mg/kg pentobarbital i.v. and maintained by isoflurane (1.5%) in a mixture of O_2_ and N_2_O (1:2). Periprocedurally antibiotics (ampicillin 1000 mg i.v. preoperatively and ampicillin 1000 mg i.m. postoperatively) and analgesics (metacam 0.2 mg/kg s.c. preoperatively and buprenorphine 0.3 mg i.m. postoperatively) were administered.

Ten surface-ECG leads (six limb leads, four precordial leads) were recorded throughout the experiment and stored on hard disk. Under aseptic conditions, the femoral artery and vein and carotid artery were dissected and sheaths were placed by Seldinger technique. Right and left ventricular monophasic action potential (RV and LV MAP) catheters (Hugo Sachs Elektronik GmbH, March, Germany) and a left ventricular 7F pressure catheter (CD Leycom Inc., Zoetermeer, Netherlands) were positioned under fluoroscopic guidance.

### Experimental Protocol

Two serial experiments were performed. In the first experiment, baseline surface ECG, LV, and RV MAPD and left ventricular pressure during SR were recorded. Subsequently, His bundle ablation was done as described previously (Schoenmakers et al., 2003). When the idioventricular rhythm (IVR) was too low, a pacemaker was implanted subcutaneously with a transvenous lead in the RV apex. At AAVB, a pacing protocol (see below) was performed and the effects on LV pressure were recorded. Next, susceptibility to TdP was tested by a pro-arrhythmic challenge with the I_Kr_ blocker dofetilide (0.025 mg/kg i.v, infused during 5 min or until the first TdP occurred). TdP was defined as a run of five or more ectopic beats, with twisting morphology of the QRS-complexes. When ≥3 TdP occurred in the first 10 min after start of dofetilide administration, the dog was considered inducible.

During the second experiment, after 2 weeks of remodeling at CAVB2, baseline ECG, LV and RV MAPD and left ventricular pressure were recorded and the pacing protocol and susceptibility test with dofetilide were repeated.

### Pacing Protocol

FFR, MR, and PESP were measured during a pacing protocol from the LV MAP catheter. The FFR protocol consisted of 3 min of steady-state pacing at three different CLs of 300 ms, 750 ms, and 1200 ms. For the MR and PESP protocol, the LV was paced with a basic CL of 600 ms, which was interrupted every 20th beat by an extrastimulus with an incremental CI ranging from 250 ms up to 800 ms, with steps of 50 ms.

### Data Analysis

#### Contractility Measures

**FIGURE 1:**
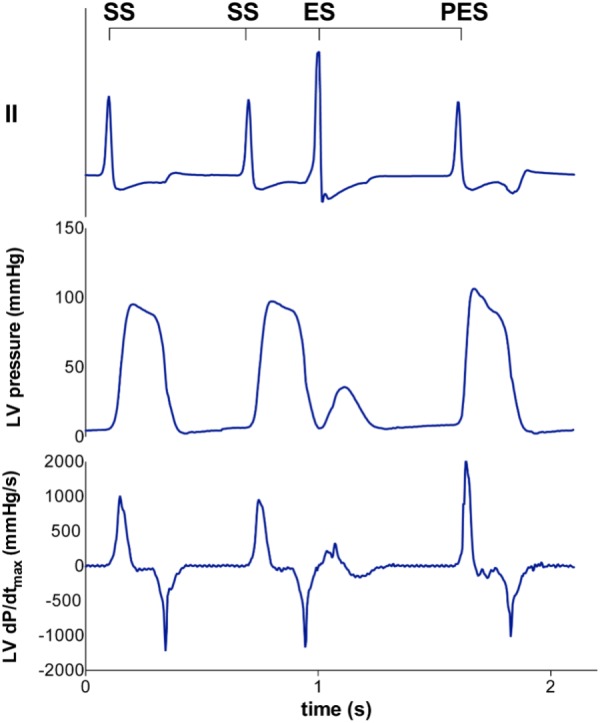
Representative tracing of LV pressure and LV dP/dt_max_ of the extrasystolic and postextrasystolic beat. A steady state stimulation train (SS) is interrupted with an extrasystolic beat (ES) at different coupling interval (CI), followed by a post-extrasystolic beat (PES) at a fixed CI. With decremental CI of the ES, the LV dP/dt_max_ of the ES decreases, while the LV dP/dt_max_ of the PES increases.

As a measure of contractility, the maximal slope of left ventricular pressure rise (LV dP/dt_max_) was calculated offline with computer software (Conduct NT, CD Leycom). For the FFR, the mean LV dP/dt_max_ of five consecutive beats was used at the three stimulation frequencies. A straight line was fitted through these points and its slope was calculated to quantify the orientation of the FFR. MR and PESP were defined as the LV dP/dt_max_ of the extrasystolic and post-extrasystolic beat, respectively (**Figure 1**). In addition, normalized MR and PESP were calculated by taking the ratio of LV dP/dt_max_ of the extrasystolic and post-extrasystolic beat, respectively, to the mean LV dP/dt_max_ of the five beats immediately preceding the extrasystole. Both MR and PESP were fitted to monoexponential functions using non-linear least squares regression, with the equation: $y=\text{a}-\text{b}*e^{-\frac{x}{TC}}$ for MR and $y=a-b*e^{-\frac{x}{TC}}$ for PESP to calculate the time constant (TC) of MR and PESP.

#### Electrophysiological Measures

Measurement of RR-interval and QT-interval was performed with calipers on lead II of the surface ECG by use of a software program (EPTracer, Cardiotek, Heilbronn, Germany). QT-interval was corrected for heart rate (QT_c_) with the van der Water formula (Van de Water et al., 1989), which gives a better estimate than Bazett formula in anesthetized dogs. LV and RV MAPD were measured from the initial peak to 80% of repolarization in a custom-made MATLAB software program. We defined interventricular dispersion (ΔMAPD) as the difference between LV MAPD and RV MAPD. STV of LV MAPD (STV) was calculated over 31 consecutive beats using the formula: $\text{STV}=\sum |D_{n+1}-D_{n}|/30\times \sqrt{2}$, where *D* represents LV MAPD. STV after dofetilide challenge was measured just prior to the first ectopic beat.

### Statistical Analysis

Data are expressed as mean ± standard error of the mean (SEM). Serial data were analyzed with a paired Student’s *t*-test or a repeated measure analysis of variance (ANOVA) with *post hoc* Bonferroni correction. Group analysis was performed with an unpaired Student’s *t*-test. Correlation analysis was done with Pearson’s correlation coefficient. All statistical analyses were performed with GraphPad Prism 6.0 (GraphPad Software Inc., La Jolla, CA, United States). A *p*-value < 0.05 was considered as statistically significant.

## Results

### Contractile Remodeling

**FIGURE 2:**
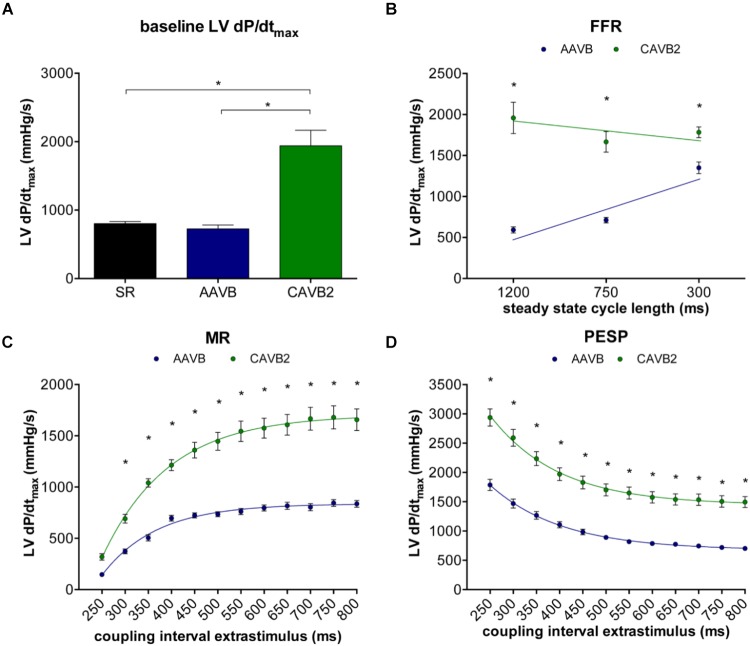
Contractile parameters at acute AV-block (AAVB) and chronic AV-block (CAVB2). **(A)** LV dP/dtmax at baseline. **(B)** Force-frequency relationship (FFR). **(C)** Mechanical restitution (MR). **(D)** Post-extrasystolic potentiation (PESP). ^∗^*p* < 0.05 compared to AAVB.

Contractile parameters at AAVB and CAVB are shown in **Figure 2**. Contractile remodeling was present after 2 weeks of CAVB, as seen by a significant increase in LV dP/dt_max_ during IVR compared to AAVB (**Figure 2A**). At AAVB, a positive FFR was observed with augmentation of LV dP/dt_max_ at higher frequencies (**Figure 2B**). This relationship was blunted at CAVB2. At AAVB, the MR curve demonstrated a monoexponential rise in LV dP/dt_max_ toward a plateau with lengthening of the CI. The PESP curve displayed the opposite behavior. (**Figures 2C,D**). Both these parameters were increased at CAVB2.

### Electrical Remodeling

The course of electrical remodeling is depicted in **Table 1**. At AAVB, all electrophysiological parameters have adapted to the sudden drop in heart rate. When challenged with dofetilide, a further significant increase was seen in QT, LV and RV MAPD, and STV. After 2 weeks of CAVB, electrical remodeling was present, as seen by a significant increase in QT, QTc, and LV MAPD under baseline conditions compared to AAVB. RV MAPD did not increase to the same extent as LV MAPD, thus creating a high interventricular dispersion (ΔMAPD). Finally, also STV showed a significant increase, which was further augmented after infusion of dofetilide.

**Table 1:** Electrophysiological parameters.

	SR	AAVB	AAVB + dof	CAVB2	CAVB2 + dof
RR (ms)	571 ± 11	1048 ± 72^∗^	1229 ± 75	1262 ± 47^†^	1452 ± 62^§^
QT (ms)	261 ± 4	327 ± 9^∗^	552 ± 22^†^	421 ± 16^†^	593 ± 19^§^
QTc (ms)	298 ± 3	319 ± 9^∗^	543 ± 30^†^	398 ± 14^†^	553 ± 19^§^
LV MAPD (ms)	193 ± 3	240 ± 6^∗^	454 ± 23^†^	302 ± 12^†^	470 ± 30^§^
RV MAPD (ms)	190 ± 14	216 ± 6^∗^	345 ± 10^†^	245 ± 6	341 ± 22^§^
ΔMAPD (ms)	9 ± 2	24 ± 5^∗^	138 ± 24^†^	56 ± 8^†^	130 ± 25^§^
STV (ms)	0.36 ± 0.02	0.72 ± 0.10^∗^	1.91 ± 0.22^†^	1.51 ± 0.31^†^	2.66 ± 0.34^§^

*^∗^*p* < 0.05 versus SR. ^†^*p* < 0.05 versus AAVB. ^§^*p* < 0.05 versus CAVB2.*

### Contractile and Electrical Remodeling and Susceptibility to TdP

**FIGURE 3:**
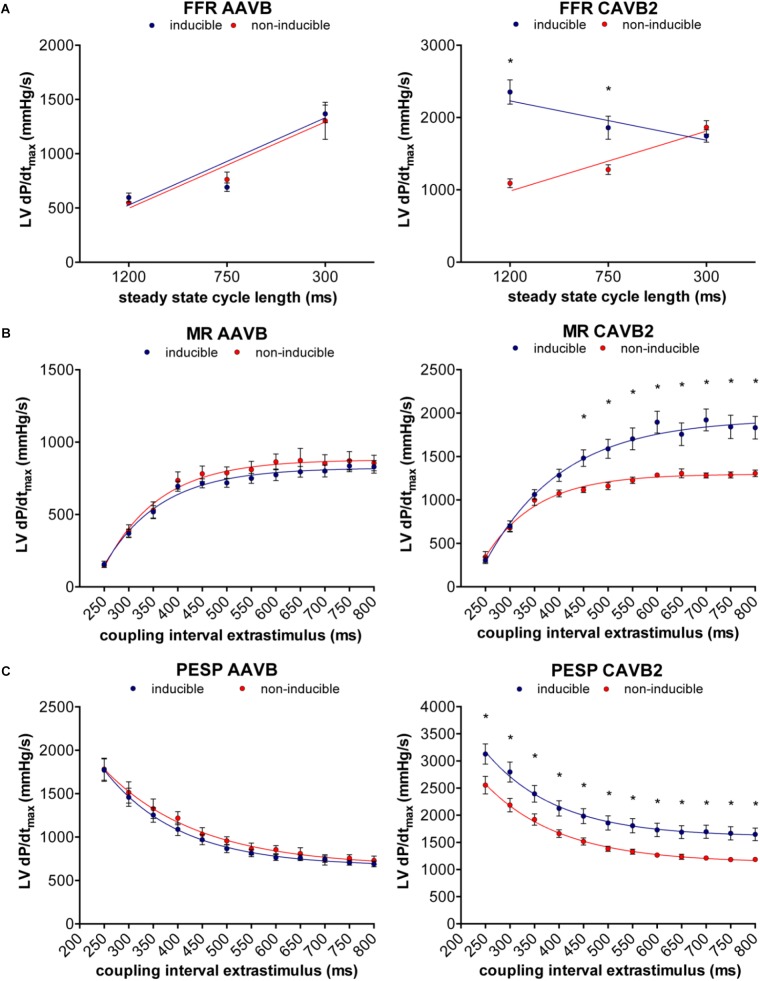
Relation between contractile remodeling and susceptibility to TdP. **(A)** FFR, **(B)** MR, and **(C)** PESP of inducible dogs (blue line) and non-inducible dogs (red line) at AAVB (left) and at CAVB2 (right). ^∗^*p* < 0.05 compared to non-inducible.

Of the 18 dogs tested, one was susceptible to dofetilide-induced TdP at AAVB. After 2 weeks of CAVB, 12/18 dogs (67%, *p* = 0.002 compared to AAVB) were inducible after dofetilide infusion. Separate measurement of contractile parameters of the inducible and non-inducible dogs are depicted in **Figure 3**. At AAVB, no differences were found in contractile measures between the two groups. However, at CAVB2, the inducible dogs demonstrated a completely different pattern of contractile remodeling compared to the non-inducible dogs. At low stimulation frequency with a CL of 1200 ms, the inducible subjects had augmentation of contractility compared to the non-inducible dogs (2354 ± 168 mmHg/s versus 1091 ± 59 mmHg/s, *p* < 0.001; **Figure 3A**). Furthermore, the slope of the FFR-curve was inverted in the inducible dogs, while the non-inducible dogs retained their positively oriented FFR (slope of -0.51 ± 0.19 in inducible dogs versus 0.89 ± 0.06 in non-inducible dogs, *p* < 0.001). As seen from **Table 2**, these differences in LV dP/dt_max_ between inducible and non-inducible dogs are not due to differences in end-diastolic pressure (EDP). A significantly higher end-systolic pressure (ESP) is seen in the inducible dogs at low stimulation rate.

**Table 2:** Hemodynamic parameters of non-inducible and inducible dogs.

		AAVB		CAVB	
		non-inducible	inducible	non-inducible	Inducible
LV EDP (mmHg)	CL 1200 ms	14 ± 2	16 ± 1	12 ± 2	11 ± 1
	CL 750 ms	14 ± 2	16 ± 1	10 ± 2	10 ± 1
	CL 300 ms	14 ± 1	15 ± 1	11 ± 3	9 ± 1
LV ESP (mmHg)	CL 1200 ms	60 ± 4	55 ± 4	73 ± 3	88 ± 2^∗^
	CL 750 ms	63 ± 9	62 ± 2	85 ± 3	85 ± 3
	CL 300 ms	69 ± 9	69 ± 4	87 ± 6	77 ± 4
LV dP/dtmax (mmHg/s)	CL 1200 ms	547 ± 10	597 ± 42	1091 ± 59	2354 ± 168^∗^
	CL 750 ms	763 ± 69	692 ± 39	1280 ± 68	1860 ± 160^∗^
	CL 300 ms	1304 ± 170	1368 ± 81	1865 ± 94	1745 ± 86

*^∗^*p* < 0.05 versus non-inducible during dofetilide infusion.*

**FIGURE 4:**
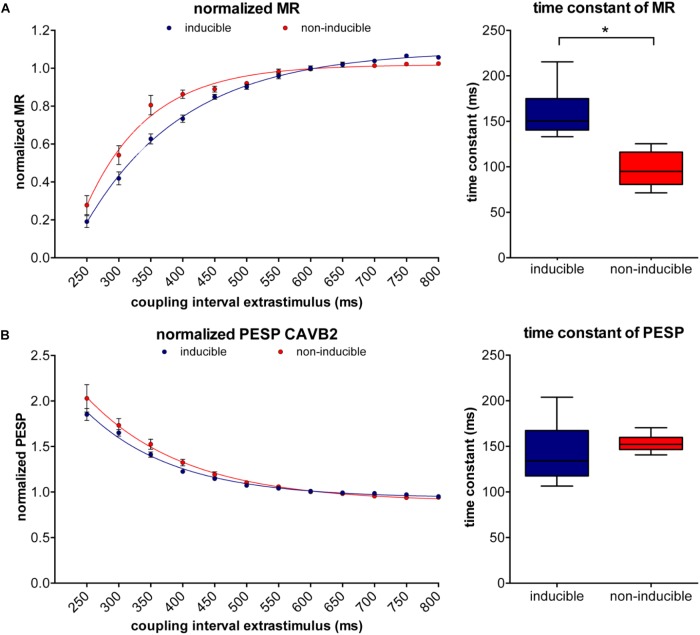
Normalized MR and PESP curves of inducible and non-inducible dogs. **(A)** The inducible dogs show slower restitution compared to the non-inducible dogs (left) with significantly higher time constants (TCs; right). **(B)** Normalized PESP curves are similar in inducible dogs and non-inducible dogs. ^∗^*p* < 0.05.

At small CIs, MR was similar in both groups, but diverged when the CI of the extrasystole rised above 450 ms, after which the susceptible dogs showed a significantly higher LV dP/dt_max_ (1483 ± 96 mmHg/s versus 1117 ± 31 mmHg/s, *p* = 0.01, **Figure 3B**). PESP, on the other hand, was increased in the inducible dogs at whole range of CIs (**Figure 3C**). When MR was normalized to the LV dP/dt_max_ of the preceding beats, the TC of MR appeared significantly higher in the inducible dogs (TC = 158 ± 7 ms versus TC = 97 ± 8 ms in non-inducible dogs, *p* < 0.001, **Figure 4A**). Normalized PESP was similar between the groups and had equal TC (143 ± 9 ms versus 153 ± 4 ms, *p* = 0.46, **Figure 4B**).

Electrically, no differences were found between the two groups at AAVB. At CAVB, the RR-interval, QT- and QTc-interval, LV MAPD, RV MAPD, and ΔMAPD were similar between the inducible and non-inducible dogs (**Table 3**). Only STV could distinguish between inducible and non-inducible dogs both at baseline and when challenged with dofetilide. Dogs that were inducible to drug-induced TdP had significantly higher STV at baseline (1.98 ± 0.41 ms versus 0.61 ± 0.08 ms, *p* = 0.009) which increased even more when challenged with dofetilide (3.18 ± 0.36 ms versus 1.35 ± 0.08 ms, *p* = 0.001).

**Table 3:** Electrophysiological parameters of non-inducible and inducible dogs.

	Baseline		Dofetilide	
	Non-inducible	Inducible	Non-inducible	Inducible
RR (ms)	1272 ± 33	1257 ± 69	1507 ± 107	1373 ± 72
QT (ms)	383 ± 19	439 ± 20	596 ± 50	587 ± 25
LV MAPD (ms)	292 ± 23	308 ± 14	494 ± 48	460 ± 39
RV MAPD (ms)	236 ± 9	249 ± 8	378 ± 53	328 ± 23
ΔMAPD (ms)	57 ± 10	56 ± 9	116 ± 14	135 ± 35
STV (ms)	0.61 ± 0.08	1.98 ± 0.41^∗^	1.35 ± 0.08	3.18 ± 0.36^†^

*^∗^*p* < 0.05 versus non-inducible in baseline. ^†^*p* < 0.05 versus non-inducible during dofetilide infusion.*

### Relation of Contractile and Electrical Remodeling With Severity of Arrhythmias

**FIGURE 5:**
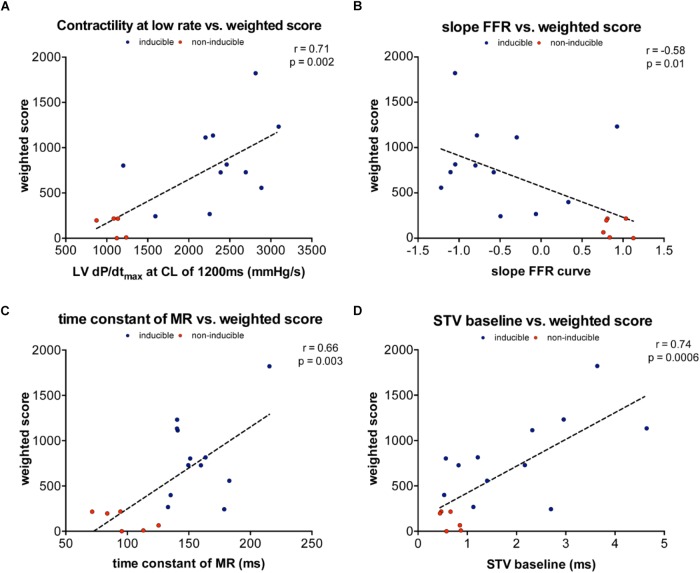
Linear correlations between weighted arrhythmia score and contractile and electrical remodeling. **(A)** Linear correlation between weighted and contractility at 1200 ms (*r* = 0.71, *p* = 0.002). **(B)** Linear correlation between weighted score and slope of FFR (*r* = –0.58, *p* = 0.01). **(C)** Linear correlation between weighted score and TC of MR (*r* = 0.66, *p* = 0.003). **(D)** Linear correlation between weighted score and STV at baseline (*r* = 0.74, *p* = 0.0006).

In order to quantify the severity of arrhythmias, we developed a new weighted scoring system, which takes into account both the number of arrhythmias in 10 min and whether the arrhythmias were sustained or defibrillations were needed (**Table 4**). This weighted score was correlated with both contractile and electrical remodeling. As illustrated in **Figure 5**, a linear correlation was found between this weighted score and LV dP/dt_max_ at CL of 1200 ms (*r* = 0.71, *p* = 0.002), the slope of FFR (*r* = -0.58, *p* = 0.01) and TC of MR (*r* = 0.66, *p* = 0.003). Of the electrical parameters, STV at baseline was also correlated with the weighted score of arrhythmic severity (*r* = 0.74, *p* = 0.0006).

**Table 4:** Custom-made weighted score of the number and severity of arrhythmias.

Arrhythmia	Points
Single ectopic beat	1
Double ectopic beats	2
Triple ectopic beats	3
Four ectopic beats	4
nsTdP (<50 complexes)	10
Sustained TdP (>50 complexes) or defibrillation	100
>1 consecutive defibrillations	200

*Score is the total number of points during 10 min after start of dofetilide infusion.*

### The Effect of Ouabain on Contractility and TdP-Inducibility

**FIGURE 6:**
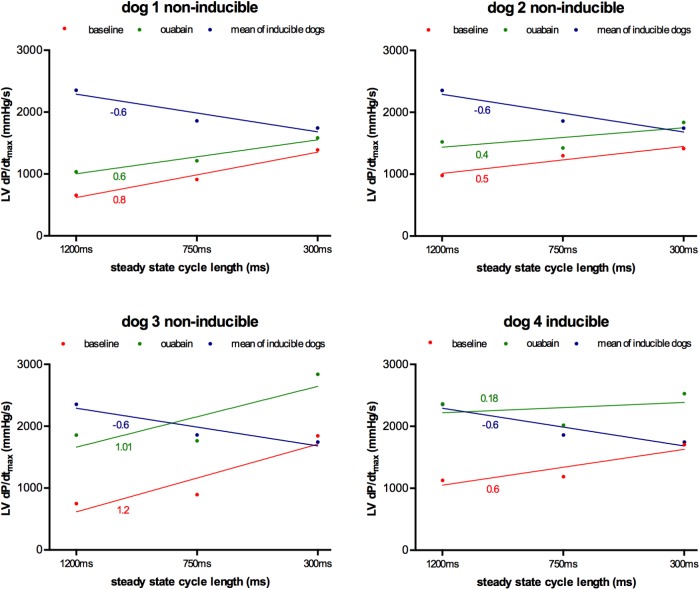
Effect of ouabain on force-frequency relationship in non-inducible subjects (*n* = 4). Contractility at CL of 1200, 750, and 300 ms at baseline (red line) and after ouabain (green line) in four non-inducible dogs. As comparison, the mean of the inducible dogs is shown (blue line). Only dog 4 became inducible after ouabain in combination with dofetilide. TCs of the different curves are given.

**FIGURE 7:**
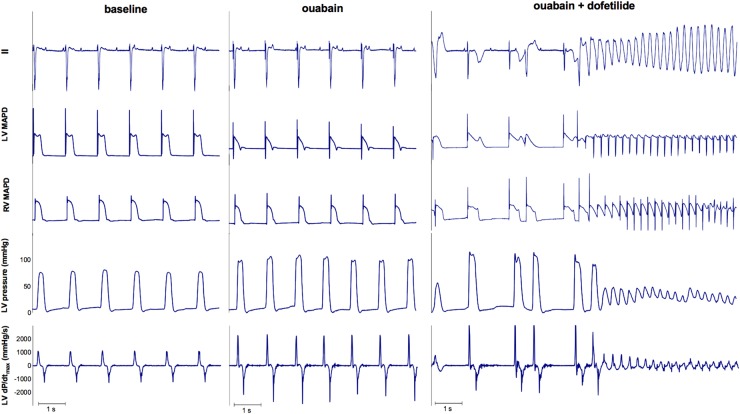
ECG, LV MAP, RV MAP, LV pressure, and LV dP/dt_max_ of dog 4. Administration of ouabain did not cause any arrhythmias. However, the combination of ouabain and dofetilide resulted in Torsade de Pointes arrhythmias.

As a proof of principle of the importance of contractile remodeling and Ca^2+^-overload for the inducibility of TdP-arrhythmias in the CAVB dog model, we attempted to pharmacologically increase Ca^2+^-load to alter FFR and potentially convert an initial non-inducible dog into an inducible one. Four non-inducible dogs were given a single dose of the cardiac glycoside ouabain (0.045 mg/kg i.v. in 1 min) prior to administration of dofetilide. In **Figure 6**, the effects of ouabain administration on FFR are depicted. In all dogs, ouabain resulted in a rise in contractility. Ouabain itself did not induce any arrhythmias. Unfortunately, in three out of four dogs, contractility at a CL of 1200 ms could not reach a level comparable to that of the inducible dogs and retained a positive FFR. As expected, these three dogs remained non-inducible after additional dofetilide infusion. Nevertheless, in one dog, contractility after ouabain administration increased to a level similar to that of the inducible dogs, with a more blunted FFR. Interestingly, this dog did become inducible to TdP-arrhythmias after infusion of dofetilide (**Figure 7**).

## Discussion

The results of this study demonstrate that contractile remodeling is already present after 2 weeks of CAVB and that this remodeling process differs substantially between TdP-susceptible and TdP-resistant dogs. Moreover, the more contractile or electrical remodeling have occurred, the higher the number and severity of arrhythmias after dofetilide challenge. Alterations in Ca^2+^-homeostasis may be the common denominator that explains the observed relation between contractile remodeling and arrhythmogenesis in the CAVB-dog model.

### Ca^2+^ Overload, DADs, and EADs in the CAVB-Dog

The present study is, to our knowledge, the first to show a direct relation between *in vivo* measurements of contractility and inducibility of TdP-arrhythmias. Nevertheless, the relation between disturbances of Ca^2+^-homeostasis and arrhythmogenesis on a cellular level is well established. Previous *in vitro* studies have demonstrated altered Ca^2+^ handling in isolated cardiomyocytes of CAVB-dogs with increased amplitude and duration of Ca^2+^-transients along with a higher Ca^2+^-content of the SR (Sipido et al., 2002). Furthermore, NCX is upregulated, which contributes to further loading of the SR via “reverse mode” Ca^2+^-influx (Sipido et al., 2000). The resulting SR Ca^2+^ overload is associated with spontaneous SR Ca^2+^ release, which can generate a DAD via “forward mode” function of NCX (exchange of 1 Ca^2+^-ion for 3 Na^+^-ions, creating an inward current) (Pogwizd and Bers, 2002). Furthermore, it has become more and more evident that altered Ca^2+^ cycling and upregulation of NCX also play a role in the formation of EADs, which has been regarded as the most important trigger of TdP arrhythmias (Volders et al., 2000; Němec et al., 2010; Horváth et al., 2015).

Our results are consistent with the study by de Groot et al. (2000) which showed that the highest LV dP/dt_max_ postpacing, which reflects an increased SR-load, was associated with the occurrence of DADs on MAP recordings. Since DADs and EADs share a common molecular mechanism, we expected to find the same relation between enhanced contractility and susceptibility of TdP-arrhythmias.

### Force-Frequency Relationship and TdP-Arrhythmias

A blunted or inverted FFR has been observed in isolated muscle preparations of failing myocardium (Pieske et al., 1995, 1999, 2002) as well as in animal models (Eising et al., 1994; Neumann et al., 1998) or patients with cardiac dysfunction (Feldman et al., 1988; Hasenfuss et al., 1994; Schotten et al., 1999). In the CAVB-dog, FFR has also shown to be inverted, with increased contractility at low heart rates (de Groot et al., 2000; Peschar et al., 2004).

In the present study we observed the inverted FFR only in the inducible dogs, while the FFR of the non-inducible dogs remained positive (**Figure 3A**). There is strong evidence that increased [Na]_i_ during diastole plays a central role in the inversion of the FFR (Mubagwa et al., 1997; Mills et al., 2007). A study on rabbit papillary muscle strips examined the effect of increased [Na]_i_ on FFR and showed that treatment with the Na^+^ ionophore monensin, which increases diastolic [Na]_i_, could convert a positive FFR into a negative FFR, mainly by increasing contractility at lower heart rates (Mubagwa et al., 1997). The high diastolic [Na]_i_ will favor Ca^2+^ influx via NCX and since the ratio of diastole to systole is highest at low heart rates, Ca^2+^ loading of the SR will be enhanced, resulting in increased contractility. Dogs with CAVB have approximately 4 mM higher [Na]_i_, compared to controls, probably due to reduced [Na]_i_-affinity of the Na^+^-K^+^-ATPase and increased Na^+^-influx via the Na^+^-H^+^-exchanger type 1 (NHE-1) (Verdonck et al., 2003; van Borren et al., 2013). However, in these studies, no dofetilide challenge was performed, therefore it is unknown whether the [Na]_i_ is higher in inducible dogs compared to non-inducible dogs.

Nevertheless, we could hypothesize that in the inducible dogs a high [Na]_i_ in combination with enhanced NCX activity results in Ca^2+^-overload, spontaneous Ca^2+^ release, and subsequent EADs and TdP-arrhythmias. Supporting this hypothesis, it has been shown that lowering [Na]_i_ by administration of the selective late Na^+^-current blocker ranolazine could reduce the number of EADs *in vitro* and TdP-arrhythmias *in vivo* (Antoons et al., 2010). In addition, blockade of NCX by SEA-0400 is effective in prevention of TdP-arrhythmias in the CAVB dog (Bourgonje et al., 2013).

As a proof of concept, we tried to increase [Na]_i_ in the non-inducible dogs by blockade of the Na^+^-K^+^-ATPase with ouabain. In contrast to the cardiac glycoside digitalis, ouabain has a fast onset of action, reaching his maximum after 5 min of administration (Fuerstenwerth, 2014). The increased [Na]_i_ caused by inhibition of the Na-K-ATPase will be exchanged for Ca^2+^, causing Ca^2+^-overload of the cardiomyocyte that is responsible for both the inotropic and potential arrhythmogenic effects. As we have shown, only the dog that could reach the contractility level of the inducible dogs and developed a more blunted FFR, became susceptible to dofetilide-induced TdP, which further illustrates the importance of [Na]_i_ and [Ca^2+^]_i_ for the initiation of TdP-arrhythmias.

### Mechanical Restitution/Post-extrasystolic Potentiation and TdP-Arrhythmias

We have demonstrated that inducible dogs have a higher MR and PESP and slower MR kinetics compared to the non-inducible dogs. In previous studies altered MR and PESP have been found in patients with hypertrophy and heart failure. (Beck et al., 1971; Merillon et al., 1979; Seed et al., 1984; Prabhu and Freeman, 1995). Both phenomena are explained by time-dependent availability of releasable Ca^2+^ due to gradual translocation of releasable Ca^2+^ from an uptake compartment to a release compartment or because of a time-dependent recovery of the SR Ca^2+^-release channel, the RyR2.

Discussion on the mechanism of slower MR in the CAVB dog remains speculative, since the kinetics of RyR2 have never been investigated. Furthermore, the precise molecular mechanism behind inactivation and recovery of RyR2 remains controversial (Bers, 2008). First, it can be hypothesized that the intrinsic gating properties of the RyR2 have been altered by the remodeling process. Secondly, changes in cytosolic [Ca^2+^] have been proposed to influence the inactivation of RyR2, referred to as Ca^2+^-dependent inactivation. It is hypothesized that increased dyadic [Ca]_i_, which is responsible for RyR2 activation, might also play a role in RyR2 inactivation (Laver, 2005). Thirdly, RyR refractoriness might be caused by “functional depletion” of Ca^2+^ of the SR after global Ca^2+^-release. Refilling of the SR during Ca^2+^-reuptake increases the sensitivity of RyR2 to cytosolic Ca^2+^ and accelerates recovery from inactivation (Szentesi et al., 2004). Since Ca^2+^ homeostasis is an interplay between RyR2, SERCA2a, and NCX, we can assume that alterations in expression and function of one of these proteins, will subsequently affect normal function of the others. Increased NCX function could result in “relatively” diminished activity of SERCA2a, by changing the ratio of Ca^2+^ extrusion versus reuptake. In this regard, a study in a transgenic mouse model showed that overexpression of phospholamban, the main inhibitor of SERCA2a, caused a reduced reuptake of Ca^2+^ in the SR, but also resulted in a higher TC of MR and increased PESP compared to control, just as we found in the inducible dogs (Hoit et al., 1999, 2000). Furthermore, in isolated cardiomyocytes, inhibition of SERCA2a could significantly slow down recovery from inactivation of RyR2 (Szentesi et al., 2004). Thus, increased NCX function, which contributes to susceptibility to triggered arrhythmias in the CAVB-dog, might indirectly influence SERCA2a and RyR2 function, and thus alter MR and PESP *in vivo*.

### Electrical Remodeling and TdP-Arrhythmias

Electrically, we found that only STV can distinguish between inducible and non-inducible subjects. This is in line with previous studies on STV in the CAVB dog model, which show that STV both at baseline and after dofetilide infusion is a more powerful predictor of drug-induced TdP arrhythmias compared to the QT-interval or LV MAPD itself (Thomsen et al., 2004, 2006, 2007). The molecular basis of STV is not fully elucidated, but have been attributed to alterations in Ca^2+^ handling. In isolated cardiomyocytes, β-adrenergic stimulation during reduced repolarization reserve (blockade of the repolarization current I_Ks_) resulted in increased cellular Ca^2+^ load and spontaneous Ca^2+^ release, which was associated with increased STV and the occurrence of DADs and EADs (Johnson et al., 2010, 2013). Buffering of Ca^2+^ by BAPTA-AM, blockade of SR Ca^2+^ release with ryanodine or inhibition of NCX by SEA0400 led to a drastic reduction of STV and eliminated all DADs and EADs (Johnson et al., 2010, 2013). A study by Antoons et al. (2015) showed that STV is highly dependent on SR Ca^2+^ release, which can modulate Ca^2+^-dependent currents making the heart more prone to EADs. These data support the hypothesis that increased STV reflects disrupted Ca^2+^-homeostasis as the underlying mechanism of EADs and TdP-arrhythmias in the CAVB-dog.

### Clinical Implications

Since the CAVB dog model is a specific model of compensated hypertrophy caused by volume overload, extrapolation to a population of patients with heart failure with reduced ejection fraction should be done with caution. Nevertheless, since contractile remodeling and arrhythmogenesis are related, contractile parameters might be used in risk stratification of ventricular arrhythmias and sudden cardiac death. In addition, patients with heart failure with preserved ejection fraction (HF-pEF) have also been shown to exhibit disrupted Ca^2+^-handling, therefore the contractile parameters investigated in this study could also be used in these patients. Recently, a study of a non-invasive measurement of PESP of blood pressure, measured via a photoplethysmographic device, showed that higher PESP was correlated with increased mortality in myocardial infarction survivors (Sinnecker et al., 2014). The cause of death was not stratified in this study, however, increased PESP may be associated with increased arrhythmia risk. The currently ongoing “EUropean Comparative Effectiveness Research to assess the use of primary prophylacTic Implantable Cardioverter Defibrillators” (EU-CERT-ICD) study evaluates the relationship between non-invasive measured PESP and the incidence of ICD-shocks in a primary prevention ICD-population. This study will give further insight if non-invasive measures of contractility could function as predictors of life-threatening arrhythmias.

### Limitations

No direct measurements of Ca^2+^ transients or SR function have been done, therefore hypotheses on mechanism of contractile remodeling and the relation with TdP-arrhythmias are based on assumptions derived from previous molecular work. Explanations other than related to Ca^2+^-handling may also be possible, such as mechano-electrical feedback, i.e., direct effects of altered loading conditions on repolarization. Secondly, LV dP/dt_max_ has important limitations as a measure of contractility, since it is also dependent on ventricular loading. While we did not measure ventricular volumes, we have shown that EDP are not different between inducible and non-inducible dogs. Therefore, we can assume the differences found in LV dP/dt_max_ are predominantly caused by changes in contractility. Finally, the relation between contractile remodeling and TdP was only measured at CAVB2, therefore no conclusion can be made on this association later in the remodeling process.

## Conclusion

In the CAVB dog model, contractile and electrical remodeling are already present after 2 weeks of AV-block and develop concomitantly with susceptibility to dofetilide-induced TdP. Furthermore, contractile parameters are altered to a far larger extent in inducible dogs, as seen by development of an augmented negative FFR, higher maximal response of MR and PESP and slowed MR kinetics.

## Author Contributions

DS did experimental work, analysis of data, and wrote the manuscript. JB delivered technical support during experiments. MS contributed to experimental concepts and design of the study. MV was responsible for organization of experiments and revised the manuscript thoroughly. All authors approved the submitted version.

## Conflict of Interest Statement

The authors declare that the research was conducted in the absence of any commercial or financial relationships that could be construed as a potential conflict of interest.

## Abbreviations

AAVB: acute AV-block
CAVB: chronic AV-block
CI: coupling interval
CL: cycle length
DAD: delayed afterdepolarization
EAD: early afterdepolarization
FFR: force-frequency relationship
MAPD: monophasic action potential duration
MR: mechanical restitution
NCX: Na^+^–Ca^2+^ exchanger
PESP: post-extrasystolic potentiation
RyR2: ryanodine receptor 2
SERCA2a: sarcoplasmic reticulum Ca^2+^-ATPase 2a
SR: sinus rhythm
STV: short-term variability
TdP: Torsade de Pointes
